# Systemic complications of rheumatoid arthritis: Focus on pathogenesis and treatment

**DOI:** 10.3389/fimmu.2022.1051082

**Published:** 2022-12-22

**Authors:** Di Wu, Yehao Luo, Tong Li, Xinyi Zhao, Ting Lv, Gang Fang, Peiqi Ou, Hongyi Li, Xiaofan Luo, An Huang, Yuzhou Pang

**Affiliations:** ^1^ Zhuang Medical College, Guangxi University of Chinese Medicine, Nanning, Guangxi, China; ^2^ School of Second Clinical Medicine, Guangzhou University of Chinese Medicine, Guangzhou, Guangdong, China

**Keywords:** rheumatoid arthritis, complications, incidence, treatment, prospects

## Abstract

As a systemic autoimmune disease, rheumatoid arthritis (RA) usually causes damage not only to joints, but also to other tissues and organs including the heart, kidneys, lungs, digestive system, eyes, skin, and nervous system. Excessive complications are closely related to the prognosis of RA patients and even lead to increased mortality. This article summarizes the serious complications of RA, focusing on its incidence, pathogenesis, clinical features, and treatment methods, aiming to provide a reference for clinicians to better manage the complications of RA.

## 1 Introduction

**Figure 1:**
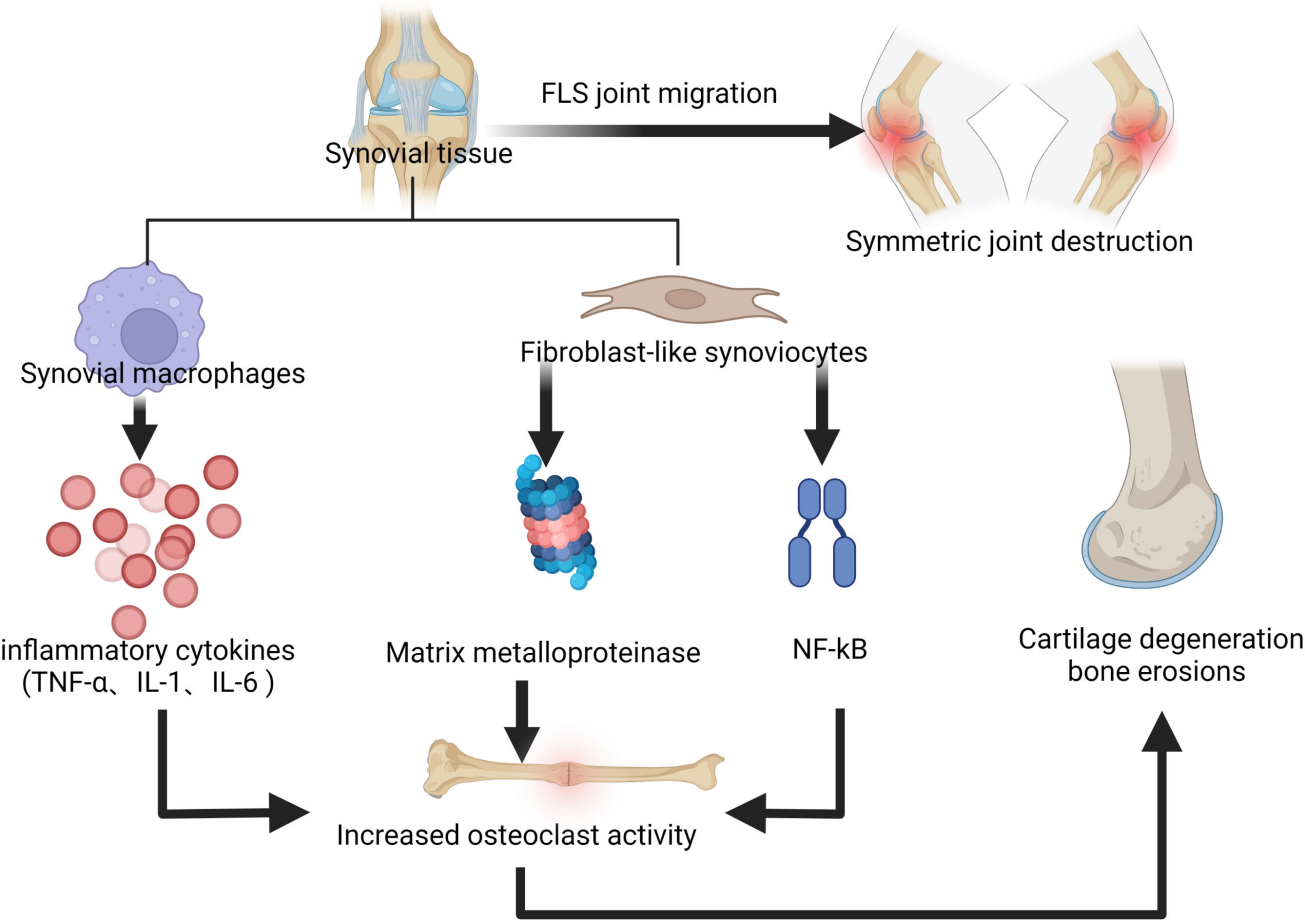
The immune processes that occur in the joint synovium and synovial fluid, leading to the progress of bone erosion and cartilage degeneration.

Rheumatoid arthritis (RA) is defined as a systemic autoimmune disease associated with a chronic inflammatory process, which gradually leads to joint destruction, deformity, disability, and even death (1). It is a widely distributed disease worldwide, with a prevalence of approximately 0.5% to 2% and a higher prevalence in women, smokers, and those with a family history of it (2). At present, the etiology of RA has not been fully elucidated, but what attracts attention is the immune processes that occur in the joint synovium and synovial fluid (3, 4), during which synovial macrophages release cytokines, such as tumor necrosis factor α (TNF-α), interleukin-1 (IL-1) and interleukin-6 (IL-6), which co-stimulate the activity of osteoclasts with inflammation and fibroblast-like synoviocytes (FLS), thus leading to the progress of bone erosion (5). In addition, activated FLS can produce matrix metalloproteinase (MMP) that leads to cartilage degeneration (6). Nuclear factor-kappa-light-chain-enhancer of activated B cells (NF-κB) is involved in the pathogenesis of chronic inflammatory diseases, and FLS stimulates the NF-κB signaling pathway, allowing T cells to bind to proteins on the surface of osteoclasts, which also leads to further development of bone erosion as it increases osteoclast activity (7). FLS can migrate from one joint to another, resulting in symmetrical joint destruction which is typical in RA (8). In addition, the presence of autoantibodies in the serum of RA patients is a mark of disease, with rheumatoid factor (RF) and anti-citrullinated protein antibodies (ACPA) being the most prominent. These autoantibodies are found in 50-80% of RA patients (9), newly-detected antibodies such as anti-carbamylated protein antibodies and anti-acetylated protein antibodies were also identified in them (10). Antibody production leads to inflammation; citrullination leads to an immune response which indicates the formation of ACPA (11); ACPA may play an important role in the prolonged inflammatory process and its presence directly links bone erosion and pain in RA patients (12). The pathogenesis of rheumatoid arthritis mentioned above is shown in **Figure 1**.

**Figure 2:**
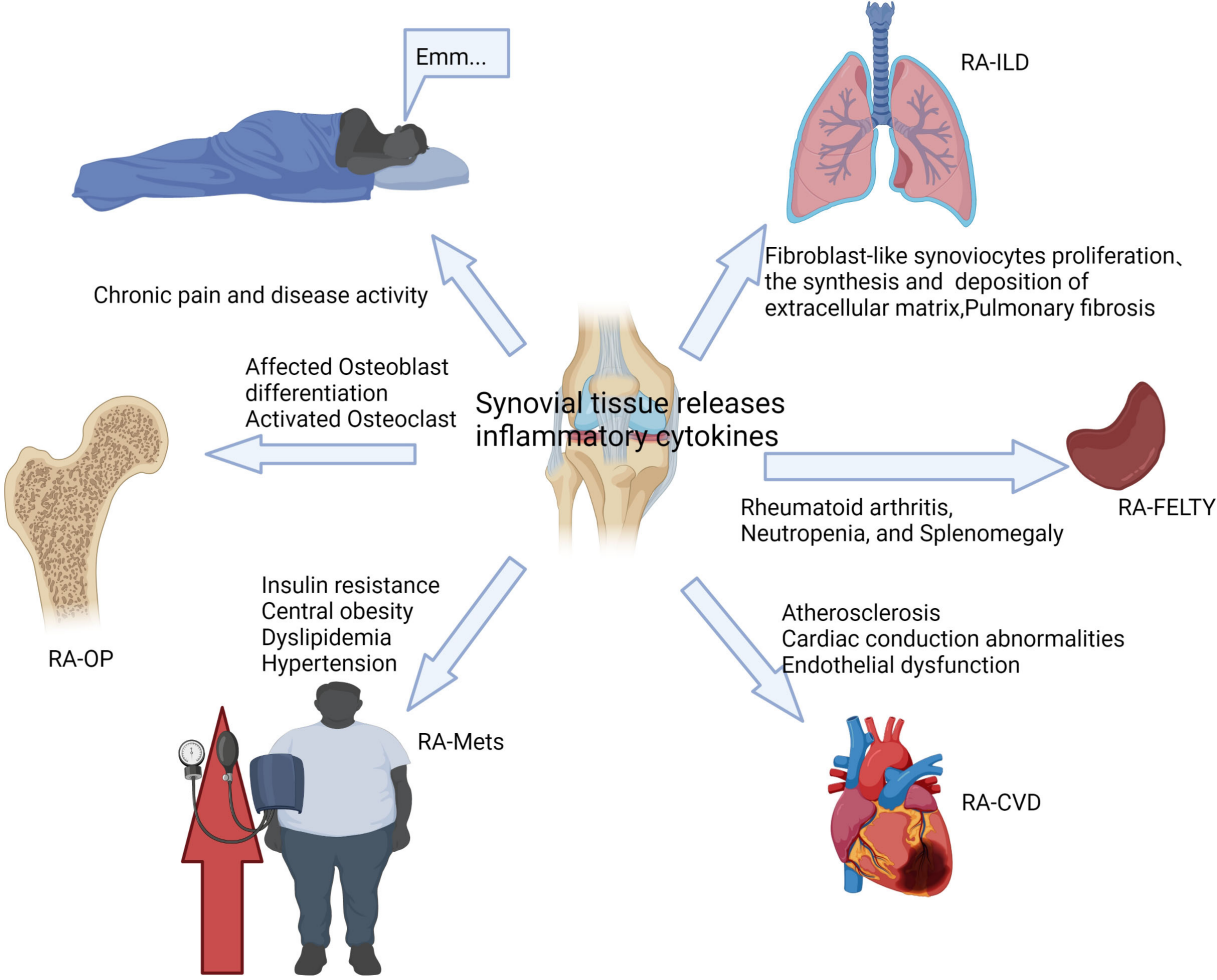
The complications of RA are usually closely related to disease activity and inflammation levels.

As a systemic disease, RA usually causes damage to other tissues and organs besides joints, including the heart, kidneys, lungs, digestive system, eyes, skin, and nervous system (13, 14). The results of the study show that about 40% of RA patients suffer from complications, and the incidence of serious complications is 8.3%, among which cardiovascular disease, interstitial lung disease, osteoporosis, and metabolic syndrome are more common (15). The existence of complications seriously reduces the quality of life of RA patients and even leads to increased RA mortality (16). Complications of RA are usually closely related to prognosis and require early diagnosis and active intervention, and the main treatment goals include reducing disease activity and controlling extra-articular damage of RA (17). At present, the treatment methods for RA complications are relatively limited. In this article, we mainly summarize the manifestations of severe extra-articular damage in RA (as shown in **Figure 2**), and discuss its pathogenesis, incidence, clinical features, and treatment methods, hoping to provide some reference for clinical practice.

## 2 Cardiovascular disease in RA

### 2.1 Pathogenesis of RA-CVD

It is well known that RA patients may be disabled, but the main cause of their death is cardiovascular disease (CVD) (18). Many studies have shown that the incidence of CVD in RA patients is 30%-60%, mainly involving pericarditis, myocarditis and heart failure, and coronary artery disease (19). Epidemiological investigations suggest that synovial tissue and circulating immune cells in RA release pro-inflammatory cytokines such as TNF-α and IL-6, which directly lead to systemic inflammation and the occurrence of CVD (20, 21). Overactive immune cells, such as T lymphocytes and B lymphocytes, may affect the cardiovascular system through multiple mechanisms (22, 23). Autoantibodies in RA affect the cascade of all structures of the cardiovascular system, from the myocardium to the heart valves, conduction system, and vasculature (24). There is more severe disease activity in ACPA-positive patients, which further leads to atherosclerosis and increases CVD mortality (25). In addition, ACPA is also seen in non-RA patients with cardiovascular disease and has adverse outcomes (26). Imaging methods are essential for the detection and assessment of CVD risk in RA, and carotid ultrasound, aortic pulse wave velocity or arterial enhancement index and ankle-brachial index, echocardiography, and cardiac magnetic resonance can be used to assess the CVD risk of patients with RA in clinical practice (27). Early detection and diagnosis of CVD in RA patients are critical for prognosis and management.

#### 2.1.1 Pericarditis

Pericarditis is one of the common cardiac manifestations of RA. Many patients with early RA can be complicated with pericarditis or develop pericarditis before RA (28). Pericarditis is inflammation and fluid accumulation in the pericardium, and about 15% of RA patients will show corresponding symptoms. However, electrocardiography shows that about 20%-50% of patients have pericardial involvement, clinically manifested as chest pain or dyspnea (29). Therefore, strict physical examination and antibody screening are needed to detect whether RA is complicated by pericarditis as soon as possible. Early diagnosis and effective treatment of pericarditis will significantly improve the prognosis of RA patients.

#### 2.1.2 Myocarditis

Myocarditis is the result of persistent inflammation in the myocardium and is histologically characterized by cellular infiltration composed of lymphocytes, histiocytes, and macrophages, which may form nodular granulomatous lesions (30). The degree of myocardial dysfunction is associated with disease activity of RA because key inflammatory cytokines in RA, such as TNFα, IL-1, and IL-6, may induce myocardial and vascular dysfunction and promote remodeling and fibrosis of the left ventricular (31).

#### 2.1.3 Arrhythmia

Arrhythmia is another common cardiac complication in RA patients, which may be secondary to conduction abnormalities. Its causes include ischemia, rheumatoid nodules, and amyloidosis (32). Recent researches indicate that symptoms and increased sympathetic nerve activity can lead to abnormal heart rhythms, and Holter monitoring can capture latent arrhythmias with higher accuracy (33).

#### 2.1.4 Coronary artery disease

The main etiology of coronary artery disease in RA may be related to atherosclerosis accelerated systemic inflammatory response and abnormal lipids and endothelial dysfunction (34, 35). The chronic inflammation and reactive oxygen species (ROS) response of RA is the core of the pathogenesis of atherosclerosis (36). ROS is a group of small active substances that play a key role in the regulation of biological cellular processes. The balance between ROS and antioxidants is critical for maintaining cellular homeostasis, thus an imbalance between oxidants and antioxidant mechanisms can lead to oxidative stress states (37). Excessive ROS may lead to vascular damage, the result of a complex cascade including oxidative modification of lipoproteins, endothelial activation, and accelerated atherosclerosis by leukocyte migration and differentiation (38). Pro-inflammatory cytokines and chemokines, as well as IL-1 and intercellular and vascular cell adhesion molecules (39), are highly expressed in atherosclerotic lesions, promote leukocyte recruitment, impair vasodilation, and induce oxidation stress and promote coagulation (40).

#### 2.1.5 Heart failure

Heart failure is the main cause of death in RA patients, and the prevalence of heart failure in RA patients is also twice as high as that in the general population, with a higher incidence in women than men in general (41). Studies have found that RA patients are more likely to develop heart failure due to diastolic dysfunction, which may be related to systemic inflammation (42). Elevated levels of c-reactive protein (CRP) and erythrocyte sedimentation rate (ESR), RF, ACPA and inflammatory cytokines may contribute to the progression of heart failure in RA (43).

### 2.2 Treatment of RA-CVD

An increasing number of evidence supports that long-term use of NSAIDs has the potential of triggering cardiovascular risks despite reductions in disease activity and some adverse CVD outcomes with conventional RA drugs (44). NSAIDs anti-inflammatory drugs exert therapeutic effects by inhibiting cyclooxygenase isoforms. The drugs inhibit prostacyclin production, leading to vasoconstriction, increased blood pressure, rupture of atherosclerotic plaques, and thrombosis, thus are thought to be the main contributor to CVD in RA (45). Non-steroidal anti-inflammatory drugs (NSAIDs), such as rofecoxib, have been the fundamental treatment for patients with osteoarthritis and other types of pain, but as controlled trials and other meta-analyses indicated an increased risk of cardiovascular problems in RA patients, rofecoxib has withdrawn from the market (46). Glucocorticoids are usually used to treat RA, mainly for short-term control of disease activity. However, glucocorticoids can aggravate hypertension or cause abnormal blood lipid levels and glucose tolerance, insulin resistance, and obesity, and promote the occurrence and development of CVD (47). Studies have shown that the use of statins in RA patients can reduce the degree of arteriosclerosis and carotid plaque formation (48). RA patients treated with at least one disease-modifying Anti-Rheumatic Drugs (DMARDs) and statins at the same time have seen reduced RA-CVD mortality of 21% (49), and disease activity was significantly decreased in those RA patients whose methotrexate (MTX) and corticosteroid regimens are added with statins, and this may indicate a clear positive effect of statins in the control of RA (50).

MTX is the “gold standard” for RA treatment because it has important immunosuppressive and anti-inflammatory effects and inhibits dihydrofolate reductase (51). Many studies have demonstrated the benefits of MTX. Approximately 25-40% of patients receiving MTX alone have seen significant improvement because MTX can play a role in improving microvascular endothelial function by reducing the degree of RA disease activity, reducing the risk of CVD in RA patients, and reducing their mortality (52). In addition, methotrexate appears to have cardioprotective properties on lipids and endothelium, in contrast to patients receiving adalimumab (53). Similarly, Hydroxychloroquine (HCQ) was found to have a protective effect on the vascular endothelium of RA patients (54), and it causes a lower cardiovascular risk in RA patients (55, 56). HCQ treatment can reduce low-density lipoprotein and Triglyceride serum values, and plays an anti-platelet aggregation role, thus it is considered to be cardioprotective (57).Tumor necrosis factor inhibitor (TNFi) therapy in RA reduces CVD risk *via* inhibition of endothelial dysfunction and slows the progression of atherosclerosis by reducing the expression of pro-inflammatory cytokines and endothelial adhesion molecules (58). In a controlled study, TNFi preparations improved myocardial inflammation and myocardial perfusion in patients with RA-CVD compared with standard disease-modifying antirheumatic drugs (59).

Recently, metabolic modulation therapy has become a research hotspot. Sirtuin 1 (SIRT1) is a sirtuin involved in a wide range of transcriptional and metabolic regulation, which may affect cell proliferation and inflammatory responses and inhibit the activation of NF-κB-dependent inflammation (60). Some SIRT1 activators, such as resveratrol, a polyphenol found in wine, have been extensively studied as SIRT activators and they exhibit potent antioxidant, anti-inflammatory and anti-cancer properties (61). Resveratrol can inhibit NF-κβ-dependent inflammatory response and its effect on RA patients is under evaluation (62). Notably, serum biochemical markers such as CRP, ESR, MMP-3, and IL-6 were also significantly reduced in resveratrol-treated patients (63). In addition, metformin and its analog phenformin are hypoglycemic drugs used in diabetic patients; although the exact mechanism of action remains unclear, their effect on AMPK (Adenosine 5’-monophosphate-activated protein kinase) can be conducive to the beneficial secondary effects of these drugs such as cutting inflammatory markers, improving lipid metabolism, and reducing experimental autoimmune arthritis based on the importance of AMPK on T cells in RA (64). In particular, metformin, as an activator of AMPK, can inhibit the migration of FLS, inhibit the expression of pro-inflammatory cytokines, and downregulate the level of inflammation in RA and its comorbidities (65).

Some pathways involve extracellular targets. Mavrilimumab is a monoclonal antibody against granulocyte–macrophage colony-stimulating factor (GM-CSF), and GM-CSF is expressed at high levels in synovial fluid and plasma as well as synovial tissue cells of RA patients. Phase I and II trials of mavrilimumab in the treatment of RA showed satisfactory safety and efficacy (66). GM-CSF emphasizes the impact of “inflammatory” pathways on arteriosclerosis and endothelial dysfunction. Based on this connection, it is expected that more potential therapeutic targets will be developed to better manage cardiovascular problems in RA patients. Recent clinical studies on RA-CVD are shown in **Table 1**.

**Table 1:** Recent clinical studies of RA-CVD.

Clinical therapeutic drug	Possible mechanism
**Statins (** 49)	Reduce the degree of arteriosclerosis and carotid plaque formation
**Methotrexate (** 51)	Inhibits dihydrofolate reductase, Reducing the degree of RA disease activity
**Hydroxy Chloroquine (** 54)	Have a protective effect on the vascular endothelium, Anti-platelet aggregation
**Resveratrol (** 63)	Exhibit potent antioxidant, Anti-inflammatory, Downregulate the level of inflammation in RA
**Metformin,Phenformin (** 64)	Affects AMPK activity,Downregulate the level of inflammation, Improving lipid metabolism
**Mavrilimumab (** 66)	Decreased leukocyte activation, Modulates immune and inflammatory processes

## 3 Lung disease in RA

### 3.1 Pathogenesis of lung disease in RA

#### 3.1.1 Interstitial lung disease

ILD is a serious pulmonary complication of RA, resulting in a 10-20% mortality in RA. Pulmonary involvement is common in RA patients, among which the occurrence of pulmonary complications is approximately 60-80% (67, 68). Clinical manifestations include interstitial lung disease, small airway disease, rheumatoid nodules, pleural effusion, pulmonary vasculitis, pulmonary fibrosis, etc (69). Although RA can involve many parts of the respiratory system, such as the airway or pleura, parenchymal lung involvement is associated with the highest morbidity and mortality (70). One diagnostic study showed that approximately 50% of RA patients had interstitial lung disease, of which only 10% had clinically significant symptoms such as cough and progressive exertional dyspnea (71), and that is because cytokine, chemotactic factor, and growth factor-mediated RA inflammatory process can promote FLS proliferation, increase the synthesis and deposition of extracellular matrix, and lead to pulmonary fibrosis (72, 73). The most common patterns of RA-ILD are usual interstitial pneumonia (UIP) and nonspecific interstitial pneumonia (NSIP) (74). There is no universal treatment guideline for RA-ILD, thus accurate screening and diagnosis of the characteristics of ILD development in RA patients is critical for future research and treatment of RA patients (75). Histological biopsy, pulmonary function tests, and high-resolution computed tomography (HRCT) are valuable tools for the diagnosis and evaluation of RA-ILD (76), and HRCT can accurately capture UIP cellular and traction bronchiectasis as well as reticular abnormalities and the “ground glass opacity” in NSIP (77).

#### 3.1.2 Pleurisy and pleural effusion

Pleurisy and pleural effusion are the most common pleural manifestations observed in RA patients, with only 3-5% of patients presenting with clinical symptoms such as cough, dyspnea, chest pain, and fever, which means the majority of RA patients with the pleural disease are with no clinical manifestations (78). In terms of pathogenesis, studies have suggested that IgG, IgE, and other antibodies contribute to the formation of immune complexes to destroy the capillary endothelium and increase the capillary permeability of the pleural cavity (79). Ultrasound-guided thoracentesis can be an important test in RA patients with pleural effusion.

#### 3.1.3 Airway involvement (bronchiolitis, bronchiectasis, and cricoarytenoid arthritis)

The prevalence of airway disease is high in RA as it affects 39% to 60% of RA patients and may involve any part of the airway, including large and distal small airways. The most common manifestations are bronchitis, bronchiectasis, and cricoarytenoid arthritis (80). Pulmonary function tests and HRCT can help diagnose airway-related diseases. Chronic inflammatory infection is the main cause of bronchiectasis in RA patients, and bronchiolitis is characterized by damage to the airway epithelium, which leads to airflow obstruction (81). Because the midline of the vocal folds is adducted, cricoarytenoid arthritis manifests as hoarseness, sore throat, dyspnea, and stridor, which are primarily due to thickening of the synovial membrane of the cricoarytenoid joint and persistent cartilage erosion (82).

### 3.2 Treatment of lung disease in RA

Treatment options for RA-ILD are complicated by the possible pulmonary toxicity of many DMARDs, but their ability to improve lung function and stabilize pulmonary symptoms has been demonstrated (83, 84). Therefore, joint and pulmonary involvement should be assessed independently for therapeutic purposes (85). The Spanish Society of Rheumatology recommends the use of abatacept and rituximab in patients with RA-ILD (86). A retrospective study showed that the use of abatacept, a costimulatory antagonist of T lymphocytes, improved ILD in approximately 88% of cases and reduced their risk of infection (87). In addition, abatacept significantly reduced lung density and fibrotic histology scores and improved ILD (88). Finally, data from a retrospective multicenter study conducted in Italy in 2020 showed that 86.1% and 91.7% of patients with RA-ILD treated with abatacept for at least 6 months had stable or increased forced vital capacity and carbon monoxide diffusing capacity, respectively, while 81.4% of patients had stable or improved chest HRCT (89). Rituximab is considered safe for the treatment of RA-ILD as evidenced by observational studies (90, 91).In addition, a large observational study of patients with RA-ILD showed that the pulmonary function of most ILD patients remained stable or improved after treatment with RTX during long-term follow-up (92). The British College of Rheumatology suggests that doctors be cautious in prescribing TNFi to patients with RA-ILD and recommends RTX for the treatment of refractory ILD (93).

Interstitial lung disease is characterized by alveolar inflammation and interstitial fibrosis, thus anti-fibrotic therapies, such as nintedanib and pirfenidone, have become the spotlight, and in fact, nintedanib and pirfenidone have been proven to slow the disease progression in patients with idiopathic pulmonary fibrosis (94, 95). In addition, tocilizumab as monotherapy can stabilize or even improve ILD (96), and as an IL-6 receptor antagonist, tocilizumab can achieve anti-fibrotic effects by blocking IL-6R, which means this treatment delivers potential benefits in RA-ILD-associated pulmonary fibrosis (97). Although there are still many challenges in practical clinical application, the efficacy and safety of anti-fibrotic agents in RA-ILD patients are still under continuous research (98, 99) for better control over RA-ILD.

In addition, non-drug conservative treatment methods, such as pulmonary rehabilitation and supplemental oxygen, can be used for aged or frail patients or those with multiple comorbidities (100). The role of pulmonary physical rehabilitation in RA-ILD is unclear, but it has beneficial effects on improving dyspnea, functional exercise capacity, and quality of life in idiopathic ILD (101). However, dyspnea and poor joint mobility in patients with ILD limit their pulmonary rehabilitation, thus patients with RA-ILD should take pulmonary rehabilitation in the early course of the disease (102). In addition, supplemental oxygen can be used as primary palliative therapy to improve the quality of life of patients with severe lung disease and reduce respiratory symptoms during daily activities (103). At the same time, smoking is a major risk factor for the progression of RA-ILD, and smoking cessation is important for RA-ILD patients (104).

Lung transplantation may be an option for end-stage RA-ILD, while only a few studies have evaluated post-transplant outcomes in patients with RA-ILD. A recent study reveals that patients with ILD of connective tissue disease (including RA) had similar rates of acute or chronic rejection after lung transplantation compared with patients with idiopathic pulmonary fibrosis, and there was no significant difference in survival (105). Lung transplantation may be an option for younger patients with advanced refractory disease but is not appropriate for patients at risk of advanced age, multiple comorbidities, immobility, and other severe extra-articular damage. Recent clinical studies on pulmonary complications in RA are shown in **Table 2**.

**Table 2:** Recent clinical studies of pulmonary complications in RA.

Clinical therapeutic drug	Possible mechanism
**Abatacept (** 87)	Interferes with T cell activation, Reduces pulmonary fibrosis
**Rituximab (** 91)	Improved pulmonary function
**Nintedanib,pirfenidone (** 105)	Reduces pulmonary fibrosis
**Tocilizumab (** 96)	Blocking IL-6R, Anti-fibrotic

## 4 Metabolic syndrome in RA

### 4.1 Pathogenesis of RA-Mets

The main features of Mets in RA patients are related to inflammation-induced RA disease activity and mainly include insulin resistance (IR), central obesity, dyslipidemia, and hypertension; these manifestations (106). The prevalence of Mets in RA patients varies widely worldwide, ranging from 14.32% to 37.83% according to different criteria (107). In addition, Mets are strongly associated with accelerated atherosclerosis development and increased CVD risk, and are considered to be characteristic pathogenesis of CVD (108). Studies have shown that IR is a fundamental feature of Mets in RA, and is directly related to the levels of IL-6, TNF-α, CRP, and ESR (109). RA-induced IR leads to increased systemic inflammatory responses and directly affects endothelial dysfunction (110). In addition, the continuous increase of macrophages in obese adipose tissue has emerged as a key link to metabolic inflammation (111). Recent studies reveal the heterogeneity of adipose tissue macrophages and their interactions with adipocytes, endothelial cells, and other immune cells in the adipose tissue microenvironment (112). Adipose tissue is a multifunctional organ that, in addition to its central role in storing lipids, secrets a variety of hormones. These various product, collectively referred to as “adipocytokines” or “adipokines”, are responsible for the immune response and mediators of inflammation (113). RA is associated with IR, dyslipidemia, and changes in the adipokines profile (114). In RA, adipocytes and their surrounding macrophages induce innate and adaptive immune cells to release proinflammatory cytokines that cause cartilage degradation and osteoblast dysregulation, thus leading to arthritic disease and Mets (115).

### 4.2 Treatment of RA-Mets

In RA patients, TNF-α is an important mediator of IR; therefore, biological therapies that block proinflammatory cytokines, such as TNF-α antagonists, can reduce CRP levels in RA patients, as well as modulate lipid metabolism and improve IR (116). The majority of patients receiving anti-TNF-α biologic therapy (eg, infliximab) were observed to have significant reductions in serum insulin levels as well as insulin and glucose indices, indicating an improvement in IR (117).

Other non-TNF-α treatments,Such as Abatacept, a novel biologic already approved for the treatment of patients with RA, interferes with T cell activation and prompts the polarization of adipose tissue macrophages from pro-inflammatory M1 to anti-inflammatory M2 phenotype, thereby reducing adipose tissue inflammation to improve insulin sensitivity (118). Based on the close relationship between IR and the levels of inflammatory factors such as IL-6, a study on the IL-6 blocker tocilizumab found that intravenous administration of tocilizumab had a rapid positive effect on IR and insulin sensitivity in RA patients. These findings suggest that IL-6 blocker has a potential beneficial effect on mechanisms associated with Mets and CVD development in RA patients (119).

The Janus kinase and signal transducer and activator of the transcription pathway (JAK-STAT) has an important pathogenic role in the development of low-grade chronic inflammatory responses leading to obesity and type II diabetes (120). Tofacitinib, the first small-molecule oral selective JAK inhibitor approved for the treatment of RA patients in 2018, can reduce IR when used alone as proved by research, which brings the therapeutic potential to the JAK-STAT pathway (121, 122).

Lowering LDL cholesterol with statins is a commonly used treatment in patients with metabolic diseases, and there is evidence that statins have a direct anti-inflammatory effect because they reduce CRP levels (123) to improve RA-Mets. Recent clinical studies on RA-Mets are shown in **Table 3**.

**Table 3:** Recent clinical studies of RA-Mets.

Clinical therapeutic drug	Possible mechanism
**Infliximab (** 117)	Blocking TNF-α, Modulate lipid metabolism
**Abatacept (** 118)	Interferes with T cell activation, Downregulate the level of inflammation
**Tocilizumab (** 119)	Blocking IL-6R, Improve insulin sensitivity
**Tofacitinib (** 121)	Decreased insulin sensitivity
**Statins (** 123)	Modulate lipid metabolism, Reduces CRP levels

## 5 Osteoporosis in RA

### 5.1 Pathogenesis of RA-OP

Osteoporosis is a common systemic skeletal disease characterized by low bone mass and degeneration of bone tissue microarchitecture that lead to bone fragility and fracture susceptibility (124). A fragility fracture is defined as a spontaneous fracture caused by minimal or no identifiable trauma and is a hallmark of OP (125). Bone erosion and systemic bone loss are typical features of RA. Systemic bone loss leads to the occurrence of OP, which is one of the main complications of RA (126). The incidence rate can reach 30% of RA patients, or even higher (127). Bone fragility in RA is caused by a combination of systemic inflammation, autoantibodies circulation, and the secretion of pro-inflammatory cytokines. Inflammatory cytokines such as TNF-α, IL-6, IL-1, and immune cell-derived cytokines undermine osteoblastogenesis while promoting osteoclastogenesis (128, 129). ACPA is a determinant of bone loss (130) as it has a direct and independent effect on osteoclasts (131). The effect may be mediated by IL-8-dependent osteoclast activation, so the bone loss is more likely to occur around joints of ACPA-positive RA patients. These factors all have a deleterious effect on bone (132).

### 5.2 Treatment of RA-OP

Teriparatide, a parathyroid hormone analog, can act as an anabolic drug by reducing osteoblast apoptosis and stimulating osteoblasts to increase bone formation with subcutaneous administration (133). The study showed that teriparatide resulted in a significantly greater increase in bone mineral density levels and a significant reduction in spinal fractures, compared with the active comparator and the anti-resorptive drug alendronate, and that was confirmed in clinical practice (134). Another study showed a significant reduction in spinal fractures in RA patients treated with teriparatide (135). Furthermore, in cases of high fracture risk, calcium and vitamin D should be supplemented with anti-osteoporotic therapy (136).

The receptor activator of NF-κB ligand (RANKL) is a key molecule in osteoclast differentiation and activation and is a potential therapeutic target for osteolytic diseases (137). Denosumab is a RANKL-specific human monoclonal antibody currently used to treat osteoporosis, osteosarcoma, multiple myeloma, and bone metastases (138). RANKL is expressed at moderate and high levels in the inflammatory state of RA patients, while denosumab can prevent the receptor activator of RANKL from binding to RANK on osteoclasts, thereby inhibiting bone resorption (139). In a phase II randomized controlled trial, the result of the combined use of methotrexate and denosumab in the treatment of RA was a significant increase in bone mineral density at the lumbar spine and hip of RA patients (140), suggesting that the combination of methotrexate and denosumab can prevent the development of bone erosions in RA (141).

TNFi is the first biological agent for RA treatment and is a key drug for inhibiting inflammation (142). Inflammatory cytokines induce osteoclast maturation and inhibit osteoblast activation to perturb bone homeostasis, thus, anti-TNF therapy can improve bone homeostasis in RA patients (143, 144). Infliximab has beneficial effects on bone metabolism in RA patients, studies on the effect of TNFi on bone loss have demonstrated that the use of infliximab can improve bone loss in RA patients (145). Another observational study indicated a lower incidence of vertebral fractures in RA patients treated with TNFi, suggesting that TNFi plays a bone-protective role in RA patients (146).

Janus kinases are a family of protein tyrosine kinases JAK1, JAK2, JAK3, and TYK2, which act on signal transducers and activators of transcription, and JAK inhibitors are approved for the treatment of RA (147). Tofacitinib, a JAK inhibitor, can regulate RANKL overexpression in the synovium by inhibiting the secretion of IL-17 and IL-6 to reduce the damage to joints caused by RA inflammation as proved by research (148). It is also proved that baricitinib can improve bone loss in RA by stimulating osteoblast function (149). The above results demonstrate that JAK inhibitors are effective therapeutics to increase osteoblast function and bone formation. Recent clinical studies on RA-OP are shown in **Table 4**.

**Table 4:** Recent clinical studies of RA-OP.

Clinical therapeutic drug	Possible mechanism
**Teriparatide (** 133)	Reducing osteoblast apoptosis, Stimulating osteoblasts to increase bone formation
**Denosumab** (139)	Affecting osteoclast differentiation, Inhibiting bone resorption
**Infliximab (** 145)	Improve bone loss
**Tofacitinib (** 148)	Inhibiting the secretion of IL-17 and IL-6, Regulate RANKL overexpression
**Baricitinib** (149)	Stimulating osteoblast function

## 6 Felty syndrome in RA

### 6.1 Pathogenesis of Felty syndrome in RA

Felty syndrome is a rare and severe extra-articular manifestation of RA, with an incidence of approximately 1% of RA patients. Typical manifestations are unexplained RA-complicated neutropenia and splenomegaly (150), and due to long-term granulocyte deficiency, patients are more prone to opportunistic infections, which results in increased mortality (151). Felty syndrome is common in RA patients with a disease history of more than 10 years while it is not uncommon that patients with short onset and atypical clinical symptoms are not diagnosed or misdiagnosed (152). The cause of peripheral blood cytopenia in Felty syndrome is not fully understood, and neutropenia is the most common symptom, which may be related to the presence of granulocyte-specific antinuclear factors (GS-ANF). It has been reported that the positive rate of GS-ANF in patients with Felty syndrome is as high as 75%, while that in RA patients is only 25% to 30% (153). At the same time, the presence of IgG-like granulocyte antibodies in the peripheral blood of patients with Felty syndrome can further destroy granulocytes and reduce their ability to phagocytose immune complexes, while T cell activation can inhibit granulocyte production (154). In addition, splenomegaly can cause thrombocytopenia, and the mechanism may be related to factors such as decreased platelet production, spleen retention, peripheral platelet depletion, and peripheral immune-mediated platelet destruction (155).

### 6.2 Treatment of Felty syndrome in RA

Treatment of Felty syndrome is supportive and is aimed at controlling underlying RA while improving neutropenia to prevent life-threatening infections (156). However, due to the lack of evidence-based medicine, most drugs are empirical (157). Granulocyte colony-stimulating factor ameliorates neutropenia by inducing the production of neutrophils and has good efficacy and tolerance by patients (158). It has been reported that a patient with a 38-year history of RA and Felty syndrome had a significant increase in absolute neutrophil counts after treatment with abatacept (159). Both MTX and leflunomide can improve joint and vascular inflammation in patients with Felty syndrome (160). Currently, the most widely used drug is rituximab, an anti-CD20 monoclonal antibody that acts against mature B cells and has been approved for the treatment of complex RA. In addition, rituximab has also been reported to successfully treat refractory neutropenia in Felty syndrome (161). Another report of Felty syndrome told that the patient’s clinical symptoms has been resolved after tocilizumab treatment, and his spleen had returned to normal size, the absolute neutrophil count had stabilized, and joint erosions had not continued to worsen (162). These case reports suggest new options for the treatment of Felty syndrome. Recent clinical studies on RA-Felty are shown in **Table 5**.

**Table 5:** Recent clinical studies of RA-Felty.

Clinical therapeutic drug	Possible mechanism
**Abatacept (** 158)	Induce the formation of neutrophils
**Methotrexate**,**Leflunomide (** 160)	Reducing disease activity
**Rituximab (** 161)	Against mature B cells
**Tocilizumab (** 162)	Reducing disease activity

## 7 Sleep disorders in RA

### 7.1 Pathogenesis of sleep disorders in RA

Sleep disorder is closely related to the development of chronic disease. In the long course of RA, chronic pain and disease activity may be the main factors related to sleep disorders in RA (163, 164). Sleep disorder is multifactorial thus the degree of disease activity increases the risk of depression and anxiety in RA patients, while depression can affect the quality of life and treatment compliance of RA patients. The above factors, which are underestimated or even ignored, all contribute to sleep disorders caused by disease activities and emotional problems (165). In fact, the incidence of sleep disorder in RA patients is as high as 50% (166), and poor sleep quality severely undermines the physical function of patients. Therefore, it is necessary to pay attention to the treatment of sleep disorders in RA patients because of their crucial impact on patients’ quality of life.

### 7.2 Treatment of sleep disorders in RA

Studies have shown that anti-TNF and other biologics can improve the sleep quality of RA patients. Abatacept significantly improves sleep disorders in RA patients as the MOS-Sleep Scale demonstrated its validity, reliability, and sensitivity to changes (167). Infliximab improves sleep quality and relieves vigilance disorders in RA patients, possibly a result of central effects by suppressing TNF-α circulation (168). In addition, adalimumab was proven to be beneficial in improving sleep disorder in RA patients for it reduces disease activity while improving sleep problems in RA patients (169). Another study has shown that the IL-6 antagonist tocilizumab improved sleep quality in RA patients, yet patients’ disease activity was not significantly reduced, which deserves further study as it seems to indicate a potential role of IL-6 in sleep regulation (170). Recent clinical studies on sleep disorders in patients with RA are shown in **Table 6**.

**Table 6:** Recent clinical studies of Sleep Disorders in RA.

Clinical therapeutic drug	Possible mechanism
Abatacept (167)	Reducing disease activity
Infliximab (168)	Inhibition of circulating TNF-α levels
Adalimumab (169)	Reducing disease activity
Tocilizumab (170)	Regulation of IL-6 levels

## 8 Conclusion

RA complications are a major scientific issue worthy of attention. However, the current international research on the pathological mechanism of RA complications remains unclear, and safe and effective clinical drugs and methods are limited. Given that much of the extra-articular damage in RA is related to disease activity and disease severity, control of disease activity in RA should be the optimal treatment, and earlier and more aggressive management of RA can reduce the impact of complications on prognosis. Although there exist some guidelines on the management of RA-related complications, the range of recommendations including ILD and CVD is still limited. In this review, we discuss the pathogenesis, morbidity, and updated management guidelines of serious complications such as cardiovascular problems and pulmonary involvement in patients with RA. We hope that the recommendations reviewed in this article can provide clinicians with a better reference to treatment options for RA complications.

## Author contributions

DW, YL designed the study together, equal contribution, Listed as co-first author. AH, YP as co-corresponding author, TLi, XZ, TLv, PO, HL, XL were all involved in the revision of the manuscript, GF, AH, YP made final critical revisions. All authors contributed to the article and approved the submitted version.
