# Supplementary material for: Systemic complications of rheumatoid arthritis: Focus on pathogenesis and treatment
Source: Front Immunol. 2022 Dec 22;13:1051082. doi: 10.3389/fimmu.2022.1051082 (PMC9817137; doi:10.3389/fimmu.2022.1051082)
Supplement: Supplementary file 1 [file DataSheet_1.docx]

Supplementary Material

## 1 Supplementary Figures


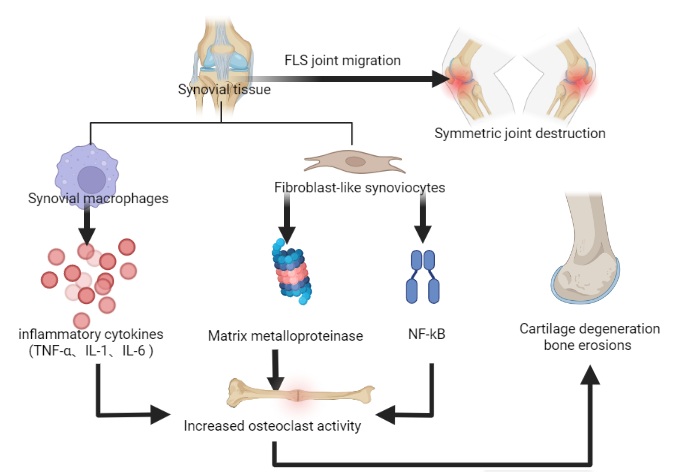


Fig 1.The immune processes that occur in the joint synovium and synovial fluid, leading to the progress of bone erosion and cartilage degeneration.


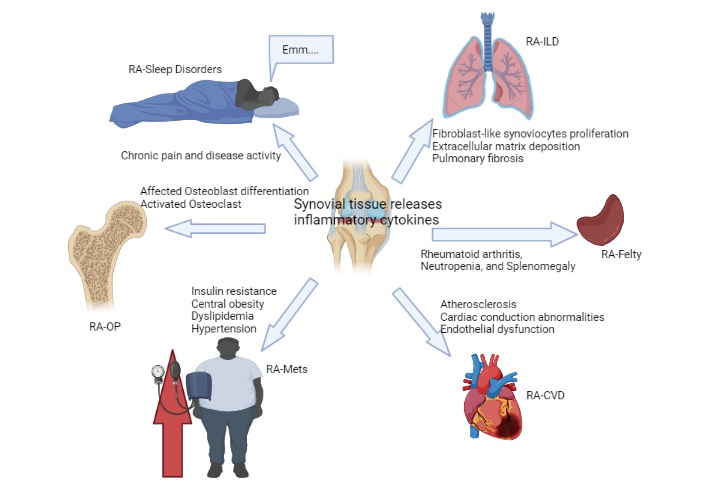


Fig 2

The complications of RA are usually closely related to disease activity and inflammation levels.

Table 1.Recent clinical studies of RA-CVD

| Clinical therapeutic drug | [Possible mechanism](javascript:;) |
| --- | --- |
| Statins^[49]^ | Reduce the degree of arteriosclerosis and carotid plaque formation |
| Methotrexate^[51]^ | Inhibits dihydrofolate reductase, Reducing the degree of RA disease activity |
| Hydroxy Chloroquine^[54]^ | Have a protective effect on the vascular endothelium, Anti-platelet aggregation |
| Resveratrol^[63]^ | Exhibit potent antioxidant, Anti-inflammatory，Downregulate the level of inflammation in RA |
| Metformin,Phenformin^[64]^ | Affects AMPK activity,Downregulate the level of inflammation, Improving lipid metabolism |
| Mavrilimumab^[66]^ | Decreased leukocyte activation, Modulates immune and inflammatory processes |

Table 2.Recent clinical studies of pulmonary complications in RA

| Clinical therapeutic drug | [Possible mechanism](javascript:;) |
| --- | --- |
| Abatacept^[87]^ | Interferes with T cell activation, Reduces pulmonary fibrosis |
| Rituximab^[91]^ | Improved pulmonary function |
| Nintedanib,pirfenidone^[94]^ | Reduces pulmonary fibrosis |
| Tocilizumab^[97]^ | Blocking IL-6R, Anti-fibrotic |

Table 3.Recent clinical studies of RA-Mets

| Clinical therapeutic drug | [Possible mechanism](javascript:;) |
| --- | --- |
| Infliximab^[117]^ | Blocking TNF-α, Modulate lipid metabolism |
| Abatacept^[118]^ | Interferes with T cell activation, Downregulate the level of inflammation |
| Tocilizumab^[119]^ | Blocking IL-6R, Improve insulin sensitivity |
| Tofacitinib^[121]^ | Decreased insulin sensitivity |
| Statins^[123]^ | Modulate lipid metabolism, Reduces CRP levels |

Table 4.Recent clinical studies of RA-OP

| Clinical therapeutic drug | [Possible mechanism](javascript:;) |
| --- | --- |
| Teriparatide^[133]^ | Reducing osteoblast apoptosis, Stimulating osteoblasts to increase bone formation |
| Denosumab ^[139]^ | Affecting osteoclast differentiation, Inhibiting bone resorption |
| Infliximab^[145]^ | Improve bone loss |
| Tofacitinib^[148]^ | Inhibiting the secretion of IL-17 and IL-6, Regulate RANKL overexpression |
| Baricitinib ^[149]^ | Stimulating osteoblast function |

Table 5.Recent clinical studies of RA-Felty

| Clinical therapeutic drug | [Possible mechanism](javascript:;) |
| --- | --- |
| Abatacept^[158]^ | Induce the formation of neutrophils |
| Methotrexate,Leflunomide^[160]^ | Reducing disease activity |
| Rituximab^[161]^ | Against mature B cells |
| Tocilizumab^[162]^ | Reducing disease activity |

Table 6.Recent clinical studies of Sleep Disorders in RA

| Clinical therapeutic drug | [Possible mechanism](javascript:;) |
| --- | --- |
| Abatacept^[167]^ | Reducing disease activity |
| Infliximab^[168]^ | Inhibition of circulating TNF-α levels |
| [Adalimumab](javascript:;)^[169]^ | Reducing disease activity |
| Tocilizumab^[170]^ | Regulation of IL-6 levels |
